# Unconjugated bilirubin is correlated with the severeness and neurodevelopmental outcomes in neonatal hypoxic-ischemic encephalopathy

**DOI:** 10.1038/s41598-023-50399-4

**Published:** 2023-12-27

**Authors:** Inn-Chi Lee, Chin-Sheng Yu, Ya-Chun Hu, Xing-An Wang

**Affiliations:** 1 Department of Pediatrics, Chung Shan Medical University Hospital, Taichung, Taiwan; 2 Division of Pediatric Neurology, Department of Pediatrics, Chung Shan Medical University Hospital, Taichung, Taiwan; 3 Institute of Medicine, School of Medicine, Chung Shan Medical University, #110, Section 1, Chien-Kuo North Road, Taichung, 402 Taiwan; 4 Division of Neonatology, Department of Pediatrics, Chung Shan Medical University Hospital, Taichung, Taiwan; 5 Department of Information Engineering and Computer Science, Feng Chia University, Taichung, Taiwan

**Keywords:** Neuroscience, Biomarkers, Diseases, Neurology

## Abstract

Unconjugated bilirubin (UB) levels during the first week after birth are related to outcomes in neonatal hypoxic-ischemic encephalopathy (HIE). Clinical Sarnat staging of HIE, brain magnetic resonance imaging (MRI), hearing outcomes, and neurodevelopmental outcomes ≥ 1 year were used to correlate UB in 82 HIE patients. The initial UB level was significantly correlated with lactic acid levels. The peak UB was higher (*p* < 0.001) in stage I (10.13 ± 4.03 mg/dL, n = 34) than in stages II and III (6.11 ± 2.88 mg/dL, n = 48). Among the 48 patients receiving hypothermia treatment, a higher peak UB was significantly (*p* < 0.001) correlated with unremarkable brain MRI scans and unremarkable neurodevelopmental outcomes at age ≥ 1 year. The peak UB were higher (*P* = 0.015) in patients free of seizures until 1 year of age (6.63 ± 2.91 mg/dL) than in patients with seizures (4.17 ± 1.77 mg/dL). Regarding hearing outcomes, there were no significant differences between patients with and without hearing loss. The UB level in the first week after birth is an important biomarker for clinical staging, MRI findings, seizures after discharge before 1 year of age, and neurodevelopmental outcomes at ≥ 1 year of age.

## Introduction

Birth asphyxia is a physiological disorder occurring in newborn infants as a result of a prolonged or profound mismatch between oxygen demand and delivery^1–4^. It may cause mild to severe neurodevelopmental disabilities. However, if the impact is moderate to severe, irreversible cerebral cell damage and death may result, leading to a syndrome called neonatal hypoxic-ischemic encephalopathy (HIE), which involves multiple organs. HIE is an important and common etiological factor implicated in neonatal death and neurodevelopmental outcomes. Therapeutic hypothermia for HIE has proven to be effective in reducing the adverse effects of asphyxia in newborns^5–7^. While it is used clinically to reduce neurological injuries secondary to HIE, there remains a 45–55% risk of death or moderate–severe disability in treated infants^5,6,8^. Therapeutic hypothermia is the standard of care for neonatal HIE, drawing increasing challenge on clinicians to make an early and accurate assessment of its likely severity^9^. Although hypothermia for moderate and severe HIE has proven effective in newborns^5,10,11^, it has not been shown to be beneficial for mild HIE^12–14^. Adjunctive tools or biomarkers for the optimal assessment of infants undergoing HIE with hypothermia therapy are needed for early diagnosis and timely treatment.

There are two distinct mechanisms involved in HIE. The first is hypoxic injury, which can cause hypoxia in the brain, particularly in susceptible brain regions such as the hippocampus, basal ganglion, thalamus, midbrain, and brain stem. This is called selective neuronal necrosis and status marmoratus of the basal ganglia and thalamus^15–17^. The second is ischemic change that causes ischemic injury by systemic hypotension or focal vessel infarction of the anterior cerebral artery, middle cerebral artery, posterior cerebral artery, and their branches.

Neonatal bilirubin levels often peak on the 2nd or 3rd day. This results from the accumulation of unconjugated bilirubin (UB) in the skin. Most infants with the indirect type present a benign course. However, it can be dangerous if acute bilirubin encephalopathy and kernicterus occur in cases of UB with > 30 mg/dL in healthy term infants^18–20^. Prolonged bilirubin toxicity can cause neurological injury and lead to cerebral palsy, dystonia, developmental delay, and hearing impairment^18^. The MRI change in prolonged bilirubin toxicity can involve the globus pallidus and less in the subthalamic nuclei^19^.

Early physiological increases in serum bilirubin levels in neonates may provide a protective antioxidant defense mechanism^21^. The possible antioxidant function of bilirubin has been found to be unaffected by hypothermia therapy^21^. Bilirubin functions as an antioxidant in vivo and is assumed to scavenge free radicals in proportion to the severity of the hypoxic injury^22^. In a mouse study, neurotoxicity and oxidative stress in mature brain slices were enhanced by UB, which abolished the neuroprotection induced by preconditioning^23^. UB has also been shown to induce impairment of hippocampal synaptic plasticity^24^. It has been suggested that bilirubin is involved in the balance between pro-oxidant and antioxidant agents in newborns, which can be disrupted during the transitional postnatal period. However, the pathogenesis remains unclear (Supplementary Table 1)^21,23,25,26^.

Although UB is a potential neuroprotective agent, its role in the pathogenesis of HIE is still noteworthy, and evaluating the potential consequences of HIE and initiating early treatment are critical for the long-term prognosis of HIE. Identifying early UB levels during the first week can help clinicians predict clinical staging and outcomes. In this study, we surveyed the initial UB level after the first admission and the peak UB level to correlate with clinical staging, magnetic resonance imaging (MRI) findings, neurodevelopmental outcomes, and hearing outcomes.

## Patients and methods

### Patients were newborns with HIE

We retrospectively reviewed neonatal HIE by reviewing patient charts in all cases. The data included clinical history of patients between 2015 to 2021 with fetal distress, metabolic acidosis, or positive-pressure ventilation immediately after birth. Chung Shan Medical University Hospital is a medical center located in the middle of Taiwan. Neonatal HIE was classified according to clinical Sarnat staging I (mild), II (moderate), and III (severe)^5,6^. Blood UB levels were measured at the time of admission and in the first week. Further examination due to HIE included a brain ultrasound, MRI, hearing test (automated auditory brainstem response (aABR), and auditory brainstem response (ABR) performed before discharge. For stage I patients, a series of head ultrasounds (HUS) were performed immediately at birth and at 1, 3, 7, and 14 days after birth. For clinical stages II and III, MRI was arranged after the patient’s condition was stable. We compared differences in blood UB at first admission and peak UB in the first week of HIE. Analysis of patients with different stages were correlated with short-term outcomes (clinical staging, hearing test, and MRI findings), seizure after discharge, and long-term neurodevelopmental outcomes at the age ≥ 1 year.

### Correlating changes in UB levels with MRI findings in HIE patients who received hypothermic therapy

To study the correlation between UB levels and MRI abnormalities, we divided the MRI findings into three groups in 48 patients with HIE and receiving hypothermia therapy. MRI was performed on hypothermic therapy patients after their clinical condition was stable. Group 1 showed unremarkable changes in brain parenchymal MRI; Group 2 had brain lesions in the parenchyma but the basal ganglion, thalamus, midbrain, and brain were spared; and Group 3 showed changes involving one of the basal ganglia, thalamus, and brain stem (midbrain, pons, lower brain stem), with or without involvement of other brain regions.

### Hearing tests before first discharge

Newborn hearing screening was performed using an automatic auditory brainstem evoked response (aABR) after birth in all newborns. ABR, otoacoustic emissions (OAE), and steady-state evoked potentials (SSEP) were performed in patients who failed aABR twice^27^. The degree of hearing loss was classified as normal (> 25 and ≤ 35 dB nHL) or abnormal, comprising mild (> 35 and ≤ 45 dB nHL), moderate (> 45 and ≤ 65 dB nHL), severe (> 65 and ≤ 90 dB nHL), or profound (> 90 dB nHL)^28,29^.

### UB levels were correlated with neurodevelopmental outcomes at the age of ≥ 1 year and with the occurrence of seizures after discharge before the age of 1 year

To assess neurodevelopmental outcomes for infants aged ≥ 1 year, we used the Bayley Scales of Infant and Toddler Development, Third Edition (Bayley-III). The cognitive and motor subscales of the Bayley-III scores were used to express neurodevelopmental outcomes. The Bayley-III score was classified as normal if both cognition and motor scores were ≥ 85, and abnormal if cognitive and motor subscales were < 85 in either cognition or motor parameters^30,31^. The occurrence of seizures after discharge was defined as seizures with or without fever occurring after discharge. The HIE patients receiving hypothermia therapy were then divided into two groups according to the peak UB level [(> 0 mg/dL and < 5 mg/dL) and (≥ 5 mg/dL)] were used to define the two groups.

### Statistical analysis

Significant differences between groups were evaluated using an independent *t*-test to compare the means of two independent groups, and a chi-squared test was used for categorical variables. The Pearson correlation coefficient was used to measure the strength of the linear associations between two variables, where a value of 1 indicated a perfect positive correlation, and *r* = − 1 indicated a perfect negative correlation. If the sample distribution was nonparametric, the Mann–Whitney U test was performed. Spearman's rho (*r*_*s*_), a non-parametric test, was used to measure the strength of the association between two variables. One-way analysis of variance (ANOVA) was used for independent variables to compare the means of three or more independent samples. Multivariate regression analysis was used to assess the variables and their correlations with the outcomes. Statistical significance was set at *p* < 0.05.

Ethical approval for the study was provided by Chung Shan Medical University Hospital’s Internal Review Board (RB #: CS2-14003) and the study was performed in accordance with relevant guidelines. Informed consent was obtained from a parent and/or legal guardian for participation in the study.

## Results

### Demographic data in newborns with HIE

**Figure 1:**
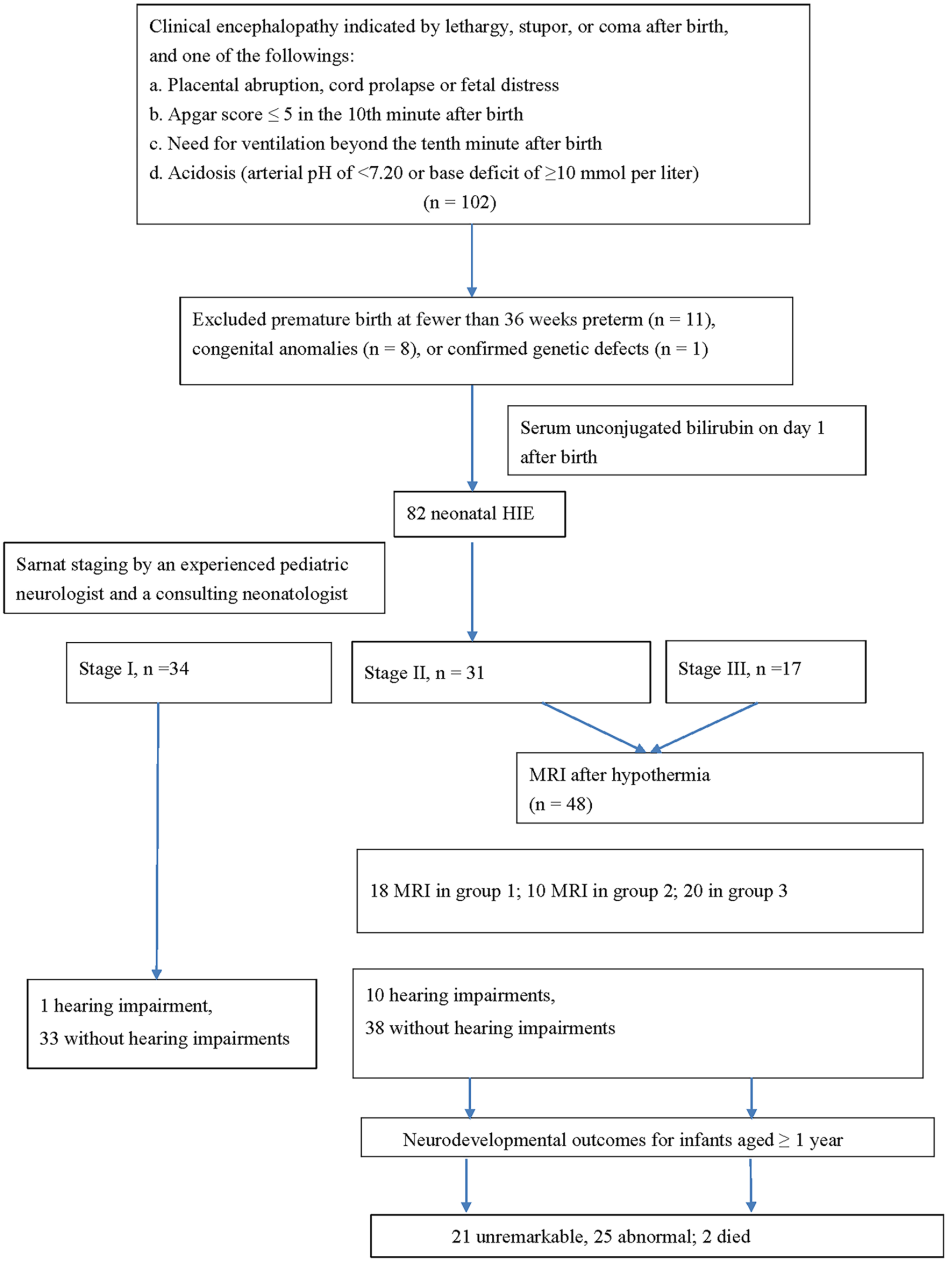
Flow chart of the 102 neonatal patients with HIE. MRI, magnetic resonance imaging.

A total of 102 HIE patients were enrolled. After excluding 20 patients due to congenital anomalies (n = 8), preterm infants with a gestational age of < 36 weeks (n = 11), or confirmed genetic defects (n = 1) (Fig. 1), a total of 82 patients with HIE remained; including 34 with stage I, 31 with stage II, and 17 with stage III HIE. Factors such as birth weight, sex, age, inborn or outborn, and method of delivery (cesarean section or vaginal delivery) were not different among the patients with stage 1, stage II, and stage III (Table 1).

**Table 1:** The demographic data in 82 patients with different clinical Sarnat staging in HIE.

	HIE stage I (n = 34)	NIE stage II (n = 31)	HIE stage III (n = 17)	*P* values among stage I, stage II, and stage III by one-way ANOVA test
Gestational age (weeks), mean (SD)	38.69 (1.21)	38.32 (1.30)	38.71 (0.92)	*F* = 0.934,* P* = 0.397
Birth weight (gm), mean (SD)	3014.9 (421.6)	2938.9(449.6)	3070.1 (535.4)	*F* = 0.491, P = 0.613
Gender				
Male	23 (67.6%)	19 (61.3%)	11 (64.7%)	*X2* (2, n = 82) = 0.287, *p* = 0.866
Female	11 (32.8%)	12 (38.7%)	6 (35.3%)	
Delivery				
Inborn	13 (38.2%)	11 (35.5%)	6(35.3%)	*X*^2^ (2, n = 82) = 0.068, *p* = 0.966
Outborn	21 (61.8%)	20 (64.5%)	11 (64.7%)	
Method of delivery				
Cesarean section	15 (44.1%)	14 (45.2%)	8 (47.1%)	*X*^2^ (2, n = 82) = 0.040, *p* = 0.980
Vaginal delivery	19 (55.9%)	17 (54.8%)	9 (52.9%)	
Apgar score at one minute, mean (SD)	5.4 (1.8)	4.3 (2.2)	2.9 (2.3)	*F* = 8.915, *P* < 0.001**
Apgar score at five minutes. mean (SD)	7.4(1.5)	5.9(2.4)	4.6 (2.4)	*F* = 11.067, *P* < 0.001**

Bold fonts indicate *p* < 0.05; **p < 0.005.

*HIE* hypoxic-ischemic encephalopathy.

### Initial UB level on day 1 correlated clinical staging

Among all HIE newborns with stage I (n = 34), stage II (n = 31), and stage III (n = 17), the initial UB level on day 1 after birth was higher (2.81 ± 2.31 mg/dL) in stage I infants than in the patients with stage II or stage III infants (2.16 ± 1.27 mg/dL, n = 48) (U = 624.5; *p* = 0.036) before hypothermia therapy.

### Correlation of initial UB level and other biomarkers of HIE

**Figure 2:**
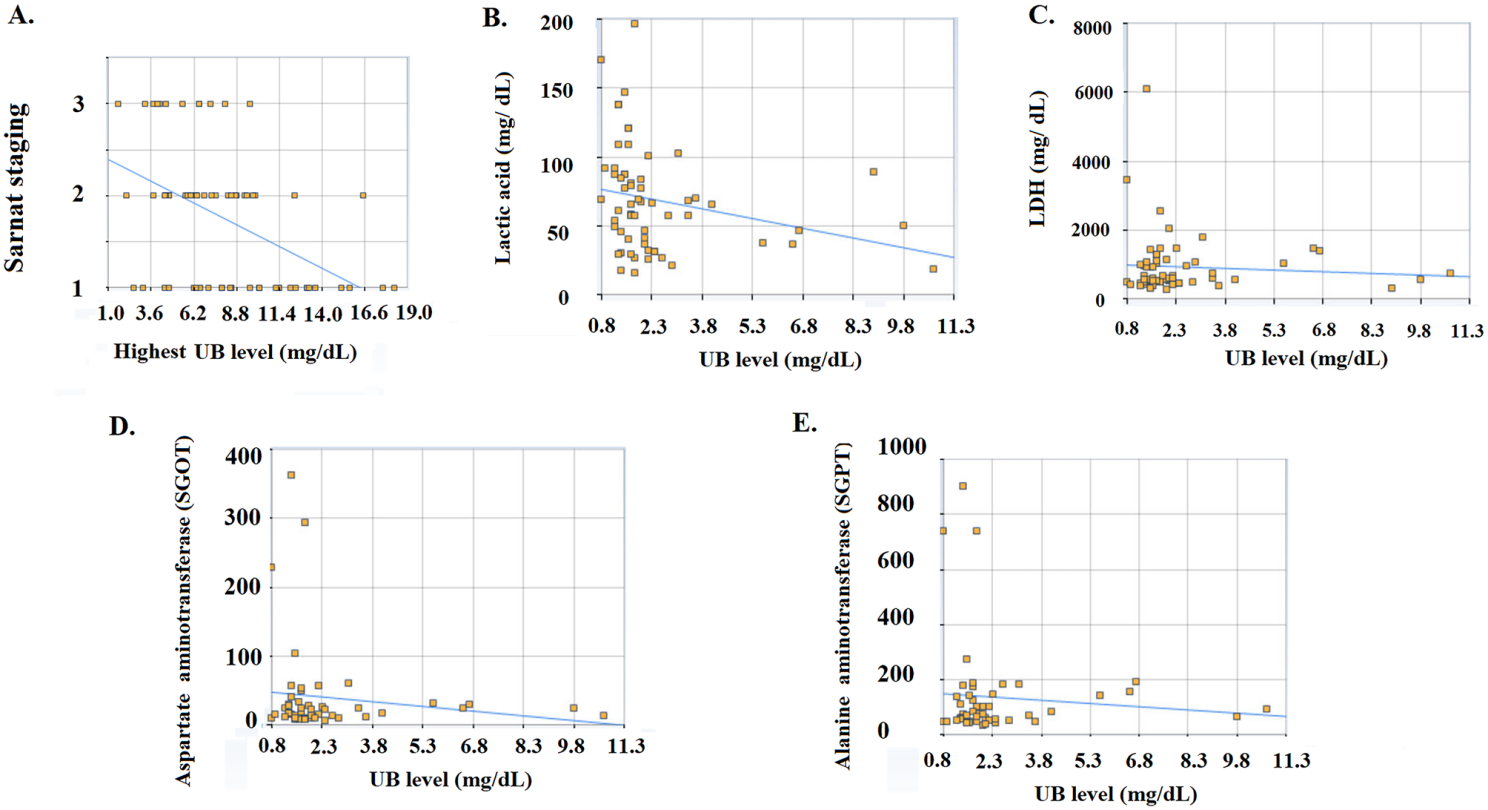
UB levels on first day was to correlate the clinical staging and systemic blood biomarkers for HIE. (**A**) clinical staging vs UB. The peak UB level was negatively correlated (*P* = 0.002) with the clinical staging. (**B**) lactic acid vs UB. Spearman's Rho calculator exhibited [Y (lactic acid) =  − 4.71X (UB*)* + 80.47] (*r*_*s*_) =  − 0.35; *p* = 0.005. (**C**) LDH vs UB. The first UB and LDH correlation was [ŷ (LDH) =  − 32.42X (UB) + 1015.6) (*r*_*s*_ = 0.092, *p* = 0.479)]. (**D**) SGOT vs UB. The first UB and GOT exhibited [ŷ =  − 7.83X + 155.6 (*r*_*s*_ = 0.077, *p* = 0.594). (**E**) SGPT vs UB. The first UB and SGPT correlation exhibited [ŷ (GPT) =  − 4.59X (UB) + 51.69 by Spearman's Rho calculator *(r*_*s*_ = − 0.082, 0.082, *p* = 0.570).

We evaluated the initial UB level and other biomarkers, including lactic acid, lactate dehydrogenase (LDH), serum liver function of glutamic oxaloacetic transaminase [SGOT], glutamic-pyruvic transaminase [SGPT]), and cardiac function of troponin I. The correlation between lactic acid and the initial UB level was evaluated using Spearman's Rho calculator as follows: [Y(lactic acid) = − 4.71X (UB*)* + 80.47] (*Spearman's* rank correlation coefficient (*r*_*s*_) = − 0.35; *p* = 0.005) (Fig. 2B). The initial UB level was significantly correlated with the initial lactic acid level in the blood. The initial UB and LDH correlation was ŷ (LDH) =  − 32.42 × (UB) + 1015.6) (*r*_*s*_ = 0.092, *p* = 0.479) (Fig. 2C). For the troponin I and UB correlation, ŷ (troponin I) =  − 7.559X (UB) + 114 using Spearman's Rho Calculator (*r*_*s*_ = 0.004; *p* = 0.975). On the first day, the UB and SGOT exhibited ŷ =  − 7.83X (SGOT) + 155.6 (*r*_*s*_ = 0.077, *p* = 0.594) (Fig. 2D). SGPT correlated with UB exhibited ŷ (SGPT) =  − 4.59X (UB) + 51.69, using Spearman's Rho calculator *(r*_*s*_ =  − 0.082, *p* = 0.570) (Fig. 2E). We found that the first day UB level was correlated with the lactic acid level immediately after admission and was not correlated with LDH, SGOT, or SGPT (Fig. 2).

### Correlation of the peak UB level during the first week with the clinical staging

Analysis of the serial UB change from day 1 to day 7 showed that the UB was still significantly higher in stage I infants than in stage II or III infants (Table 2). The peak UB in 82 infants with HIE was 7.78 ± 3.92 mg/dL. The peak UB level during the first week in the stage I group was 10.13 ± 4.03 mg/dL, which was significantly higher than that in stage II and III patients (6.11 ± 2.88 mg/dL, n = 48) (U = 331.5; *p* < 0.001). The peak UB in the first week was significantly different between stages I and II (*p* = 0.002), and between stages I and III (*p* < 0.001) (Fig. 2A and Table 2). When comparing these differences with the coefficients of variation (CV), we found that the observed differences in peak UB (stage 1, 10.13; stage 2,6.74; and stage 3:4.98) between the different stages were greater than the CV (stage 1,40.42; stage 2,45.35; and stage 3:43.17). These findings suggest that peak UB is significantly related to the three stages. The peak UB level during the first week was significantly higher in stage I (10.13 ± 4.03 mg/dL) patients than in stage II (6.74 ± 3.07 mg/dL; *p* = 0.002), and stage III patients (4.98 ± 2.15 mg/dL; *p* < 0.001) (Table 2). Bilirubin kinetics in the first week were higher in stage I, followed by stages II and III (Table 2).

**Table 2:** The serum unconjugated bilirubin on the first day after admission and in first week*.*

	HIE stage I, (n = 34), mean (SD)	HIE stage II, (n = 31), mean (SD)	HIE stage III, (n = 17), mean (SD)	**HIE** stage II + III, (n = 48), mean (SD) (*P* value in stage I vs stage II + III)	*P* values among stage I, stage II, and stage III by one-way ANOVA test
UB day 1	2.81 (2.31)	2.51 (1.45)	1.52 (0.45)	2.16 (1.27), U = 624.5, ***P* = 0.036**	*F* = 3.10, *P* = 0.050
UB day 3	9.59 (3.34)	6.01 (2.21)	5.21 (1.65)	5.80 (2.07), U = 36.0, ***P* = 0*.*015**	*F* = 7.25,* P* = 0.003** *Post-hoc*
UB day 4	9.09 (3.2)	6.93 (2.27)	5.21 (2.31)	6.23 (2.40), U = 187, *P* = 0.001**	*F* = 6.78, *P* = 0.003** *Post-hoc*
UB day 6	9.09 (4.51)	5.33 (4.68)	4.04 (3.27)	4.76 (4.10), U = 112, *P* = 0.001**	*F*** = **5.88, ***P* = 0.005** *Post-hoc*
Peak UB during first week of life	10.13 (4.03)	6.74 (3.07), **(*P* = 0**.002)** (Stage I vs. Stage II)	4.98 (2.15), (*P* < 0.001)** (Stage I vs. Stage III)	6.11 (2.88), U = 331.5, *P* < 0.001**	*F*** = **15.70, *P* < 0.001**^%^

Bold fonts indicate *p* < 0.05; **p < 0.005.

^%^Post-hoc Tukey test demonstrated Q = 4.70, *P* = 0.004 between stage I and stage II; Q = 7.15, *P* < 0.001 between stage I and stage III.

*HIE* hypoxic-ischemic encephalopathy, *UB* unconjugated bilirubin, *SD* standard deviation.

### Correlation between brain lesions in the parenchyma with peak UB levels in the hypothermia group

Among the 48 patients receiving hypothermia therapy for stage II or III HIE, MRI showed no lesions in brain parenchyma in 18 (37.5%) (group 1), 10 (20.8%) (group 2), and 20 patients (41.7%) (group 3). We found that the peak UB during the first week after admission was significantly negatively correlated with patients with positive MRI findings (*rs* = − 0.4821, *p* < 0.001) using Spearman's Rho Calculator. Among the patients with stage II or III HIE (n = 48) undergoing hypothermia therapy, those in group 1 had a higher (*F* = 14.74, *p* < 0.001) peak UB level (7.93 ± 2.95 mg/dL) compared with groups 2 and 3 (5.03 ± 2.25 mg/dL) (Table 3). When comparing these peak UB differences with the CV, we found that the observed differences in UB between different MRI results (unremarkable MRI: 50.18; abnormal MRI: 42.76) were greater than the CV (unremarkable MRI: 7.93 mg/dL; abnormal MRI: 5.03 mg/dL). These findings suggest that peak UB was significantly correlated with MRI findings in the hypothermia group. The peak UB was higher in group 1 (7.93 ± 2.95 mg/dL) than in groups 2 (5.12 ± 2.81) (*F* = 6.01, *p* = 0.021 between group 1 and group 2) and 3 (4.98 ± 2.00) *(F* = 13.22, *p* < 0.001 between group 1 and group 3) (Table 3).

**Table 3:** The peak UB level and the outcomes in stage II and stage III patients (n = 48) with HIE receiving hypothermia therapy.

	*UB* level at first day, mean (SD) (*P* values by one-way ANOVA test)	Peak UB level in first week, mean (SD) (*P* values by one-way ANOVA test)
MRI finings^★^		
Unremarkable MRI (Group 1) (n = 18) in stage II and III	2.44 (1.67)	7.93 ( 2.95)
Abnormal MRI (Group 2 + 3)	1.90 (1.01) (U = 253.0, *P* = 0.363) (Unremarkable MRI vs. abnormal MRI)	5.03** (**2.25**) **(*F* = 14.74**, *P* < 0.0010**) (Group 1 vs. Group 2 and 3)**
Group 2 (n = 10)	2.53 (1.19)	5.12 (2.81) *(F* = 6.01, ***P* = 0.021**) (Group 1 vs. Group 2)
Group 3 (n = 20)	1.73 (0.73)	4.98 (2.00) *(F* = 13.22, ***P* < 0.001**) (Group 1 vs. Group 3)**
Neurodevelopmental outcomes ≥ 1 year (n = 48)		
Unremarkable (n = 21))	2.58 (1.70)	7.41 (3.24)
Abnormal (n = 27)^**#**^	1.84 (0.70) (*F* = 4.16, ***P* = 0.047**) (Unremarkable vs. abnormal)	5.11** (**2.12**) *(****F* = 8.79**, *P* = 0.004**) (Unremarkable vs. abnormal)**
Hearing in all HIEs (n = 82)		
Normal (n = 70)	2.20 (1.36)	7.35 ± 3.97
Abnormal (n = 12)	1.49 (0.45) *(F* = *2.13, P* = 0.150) (Normal vs. abnormal)	5.74 ± 2.54 *(F* = 2.52, *P* = 0.117) (Normal vs. abnormal)
Hearing in hypothermia group		
Normal (n = 38)	2.33 (1.54)	6.08 ± 3.10
Abnormal (n = 10)	1.37 (0.32) *(F* = *2.65, P* = 0.113) (Normal vs. abnormal)	5.74 ± 2.54, (*F* = 0.137, *P* = 0.713) (Normal vs. abnormal)
Seizure after discharge		
Without seizure (n = 38)	2.24 (1.26)	6.63 (2.91)
With at least one seizure (n = 10)	1.88 (1.38) (*F* = 0.61, *P* = 0.439) (With seizure vs. without seizure)	4.17** (**1.77**)** (*F* = 6.42, ***P* = 0.015**) (With seizure vs. without seizure)

Bold fonts indicate *p* < 0.05; **p < 0.005.

*HIE* hypoxic-ischemic encephalopathy, *UB*, unconjugated bilirubin, *SD* standard deviation, *MRI* magnetic resonance imaging.

^**#**^Including 2 death before 1 year of age.

^★^Group 1 indicates unremarkable changes in brain parenchymal MRI; Group 2, brain lesions in the parenchyma but the basal ganglion, thalamus, midbrain, and brain were spared; Group 3, MRI changes involving one of the basal ganglia, thalamus, and brain stem, with or without involvement of other brain regions.

### Hearing impairments and UB levels among patients who underwent hypothermia therapy

There were 10 HIE patients in the hypothermia group having hearing impairment. Amongst these patients, the peak UB level was 5.74 ± 2.54 mg/dL and lower compared with 6.08 ± 3.10 mg/dL in those without hearing impairment; the difference was not significant (F = 0.137, p = 0.713). In all HIE (n = 82) patients, the peak UB were 7.35 ± 3.97 in those patient without hearing impairments, and 5.74 ± 2.54 mg/dL in those patients with hearing, the difference was not significant (F = 2.52, P = 0.117).

### The neurodevelopmental outcomes and peak UB levels in the HIE stage II and stage III patients who received hypothermic therapy

Among the 48 patients undergoing hypothermia, neurodevelopmental outcomes were observed in those at least 1 year old, resulting in unremarkable outcomes in 21 (43.8%) patients, and abnormal neurodevelopmental outcomes in 27 (56.2%). Analysis of the serial UB levels showed that the initial UB was significantly (*F* = 4.16, *p* = 0.047) higher in the unremarkable group (2.58 ± 1.70 mg/dL) than in the abnormal group (1.84 ± 0.70 mg/dL). When comparing these peak UB differences with the CV, we found that the observed differences in peak UB between neurodevelopmental outcomes (unremarkable group: 7.41 and abnormal group: 5.11) were greater than those in the CV (unremarkable group: 41.29 and abnormal group: 49.42). The peak UB level was significantly (*F* = 8.79**,** *p*** = **0.004**)** higher in the unremarkable group (7.41 ± 3.24 mg/dL) than in the abnormal group (5.11 ± 2.12 mg/dL). Linear regression analysis showed that UB levels were positively correlated (*p* < 0.001) with neurodevelopmental outcomes in patients at least 1 year old (Table 3).

### Occurrence of seizure before the age of 1 year after discharge in the hypothermia group

Ten (20.8%) of the 48 patients who received hypothermia therapy experienced at least one seizure after discharge, and 38 (79.2%) did not have a seizure before 1 year of age. Analysis of the serial UB levels showed that the initial UB level was insignificantly (*F* = 0.61, *p* = 0.439) higher in patients who experienced no seizures after discharge (2.24 ± 1.26 mg/dL) than in those who experienced seizures (1.88 ± 1.38 mg/dL). When assessing the differences in peak UB levels between the two groups (experienced no seizures: 6.63; and experienced seizures: 4.17), we found that these differences were greater than the differences in the CV between groups (group without seizures: 47.57; and group with seizures: 45.45). These findings suggest that peak UB levels are significantly correlated with seizures after discharge in the hypothermia group. The peak UB level was significantly (*F* = 6.42, *p* = 0.015) higher in patients who experienced no seizures after discharge **(**6.63 ± 2.91 mg/dL) than in patients who experienced seizures (4.17 ± 1.77 mg/dL)**.** Peak UB levels were negatively correlated with the occurrence of seizures before the age of 1 year. Patients with a peak UB ≥ 5 mg/dL had significantly better outcomes in brain MRI findings (*X*^2^ (1, n = 48) = 11.06, *p* < 0.001) regarding neurodevelopmental outcomes ≥ 1 year (*X*^2^ (1, n = 48) = 7.89, *p* = 0.005) compared with those with a peak UB < 5 mg/dL and those who had a seizure before the age of 1 year (***X***^2^ (1, n = 48) = 4.17, *p* = 0.041) (Table 4).

**Table 4:** The peak UB to correlate with the MRI findings, neurodevelopmental outcomes ≥ 1 year, hearing, and seizure after discharge in 48 neonatal HIEs receiving hypothermia therapy.

	Peak UB < 5 mg/ dL (n = 20) (100%)	Peak UB ≥ 5 mg/ dL (n = 28) (100%)	*P values*
MRI findings^★^			
Unremarkable MRI (Group 1) (n = 18) in stage II and III	2 (10.0%)	16 (57.1%)	*X*^**2**^ (1, n = 48) = 11.06, *p* < 0.001**
Abnormal MRI (Group 2 + 3)	18 (90.0%)	12 (42.9%)	
Group 2 (spared basal ganglion, thalamus, midbrain) (n = 10)	6	4	
Group 3 (basal ganglion, thalamus, midbrain) (n = 20)	12	8	
Neurodevelopmental outcomes ≥ 1 year (n = 48)			
Unremarkable in stage II and III (n = 21))	4 (20.0%)	17 (60.7%)	*X*^**2**^ (1, n = 48) = 7.89, ***p* = 0.005**
Abnormal (n = 27)	16 (80.0%)	11 (39.3%)	
Hearing in hypothermia group			
Normal (n = 38)	14 (70.0%)	24 (85.7%)	*X*^2^ (1, n = 48) = 1.75, *p* = 0.186
Abnormal (n = 10)	6 (30.0%)	4 (14.3%)	
Seizure after discharge			
Without seizure (n = 38)	13 (65.0%)	25 (89.385.7%)	*X*^**2**^ (1, n = 48) = 4.17, ***p* = 0.041**
With at least one seizure (n = 10)	7 (35.0%)	3 (10.714.3%)	

^★^Group 1 showed unremarkable changes in brain parenchymal MRI; Group 2 had brain lesions in the parenchyma but the basal ganglion, thalamus, midbrain, and brain were spared; and Group 3 showed changes involving one of the basal ganglia, thalamus, and brain stem (midbrain, pons, lower brain stem), with or without involvement of other brain regions.

Bold fonts indicate *p* < 0.05; **p < 0.005. MRI indicates magnetic resonance imaging.

^**#**^Including 2 death before 1 year of age.

### Multivariate regression models incorporating UB levels, lactic acid, and LDH to analyze the relationship between clinical stage and the outcomes in neonatal HIE

#### Multivariate regression models for the correlation of clinical stage in all patients

We analyzed the levels of UB, lactic acid, and LDH on day 1 after birth to determine their correlation with clinical stage in all patients. The results indicated a moderately significant relationship between umbilical bilirubin levels on day 1, lactic acid levels, LDH levels, and the clinical stage (F = 28.93, *p* < 0.001). Furthermore, a strong and significant correlation was observed between peak umbilical bilirubin levels in the first week after birth, lactic acid levels, LDH levels, and clinical staging (F = 14.32, *p* < 0.001).

#### Multivariate regression models for the correlation of clinical stage with outcomes in patients receiving hypothermia

The levels of umbilical bilirubin on day 1, lactic acid, and LDH after birth were analyzed to determine their correlation with the outcomes. The results indicated a moderately significant collective effect on neurodevelopmental outcomes after 1 year (F = 3.01, *p* = 0.092) and the occurrence of seizures after discharge (F = 9.8, *p* = 0.004). However, no significant correlations were observed with the MRI findings (F = 1.86, *p* = 0.182).

The peak UB in the first week, along with lactic acid and LDH levels, revealed a weak collective significant effect with MRI findings (F = 5.15, *p* = 0.028), a moderate collective significant effect with the occurrence of seizures before 1 year (F = 6.24, *p* = 0.004), and a moderate collective significant effect with neurodevelopmental outcomes (F = 6.24, *p* = 0.004).

## Discussion

A significant contribution of this study is its delineation of a peak UB level correlated with clinical stage, imaging findings, and neurodevelopmental outcomes at the age ≥ 1 year; however, no correlation was observed with the systemic biomarkers of LDH, liver function, and troponin in patients with HIE. In stage I HIE, the UB levels were significantly higher than in stages II and III before hypothermia treatment. The UB levels were higher in the first week after birth in stage I than in stages II and III. In the hypothermia group, we also found that the UB levels were correlated with MRI findings and neurodevelopmental outcomes at ≥ 1 year of age. Our findings suggest that bilirubin can serve a potential neuroprotective role against selective necrosis in susceptible brain tissues, including the basal ganglion, thalamus, and brain stem.

Bilirubin can exhibit both neuroprotective and neurotoxic effects on the brain^18–26^, probably due to its potential neuroprotective effects against oxidative stress. In contrast to the protective properties of UB, HIE can repress heme oxygenase^23^; however, this requires further clarification. A possible factor is that in severe HIE, patients received phenobarbital for seizure control, which also reduced UB levels^22^. In our case series, none of the patients in the hypothermia group required phototherapy due to decreased UB levels. The cause-and-effect evidence of lower UB levels may be attributed to the systemic suppression of heme metabolism by HIE or hypothermia therapy, which impairs bilirubin metabolism. However, in patients with severe abnormalities (detected using MRI) involving the basal ganglion and thalamus, decreased UB level may not explain the repression of heme oxygenase because some cases had no significant systemic effects. Selective cell necrosis caused by HIE in critical brain regions, such as the basal ganglion, thalamus, mid-brain, and brain stem, reflects lower UB levels and may result in the loss of the protective role of UB within critical regions in the brain. This finding is supported by the fact that abnormalities in the midbrain and brain stem are usually absent in patients with bilirubin encephalopathy^32^. Ischemic injury can cause focal cerebral arteriopathy or watershed infarcts. Ischemic injury causes systemic effects in multiple organs and is often associated with the elevation of systemic biomarkers such as LDH^33^, liver function^34^, and troponin^35^ in patients with HIE.

MRI scans of patients with bilirubin encephalopathy tend to differ from the MRI scans of patients with severe HIE involving the basal ganglion and thalamus^19,36^. In neonates with severe HIE, particularly stage III, usually the basal ganglion and thalamus are affected, and the midbrain and brain stem are often involved. Neurological sequelae such as dystonia, opithotonis, and asymmetrical postutes, are similar to bilirubin encephalopathy. However, in severe HIE, the consequences, including feeding and respiration, are more severe. Bilirubin in physiological concentrations protects newborns against oxidative stress in a dose-dependent fashion. However, the bilirubin concentrations of 30 mg/dL or higher can cause a significant erythrocyte cytotoxicity^37^. In a four case series^32^, the bilirubin levels were 39–50 mg/dL, and the patients developed kernicterus encephalopathy. The MRI exhibited a basal ganglion lesion, but the midbrain and brain stem were spared. We assumed that normal jaundice is necessary and has potential neuroprotective effects. A range lower or higher than the normal range indicates the potential loss of neuroprotection or toxicity to the brain, such as kernicterus. In an in vitro study, low levels of bilirubin enhanced hypoxia effects in immature neurons by facilitating glutamate-mediated apoptosis through the activation of N-methyl-D-aspartate receptors^38,39^. Suppression of hema metabolism by HIE or hypothermia cannot explain the findings related to the UB level in our study. Although the role of bilirubin as an antioxidant and scavenger of reactive oxygen species is well documented in in vitro and in vivo animal studies^21,23^, its role in newborns with or without jaundice is still debated. Carlo et al. recently showed that infants with moderate-to-severe HIE present lower peak and mean total serum bilirubin values than control infants, and speculated that this might be due to the hypoxic repression of heme oxygenase (HO)^23^. These results seem to exclude the possibility that increased levels of total serum bilirubin may act as neuroprotective antioxidant agents in infants with NE and, conversely, may cause infants who have suffered HIE further brain injury due to hyperbilirubinemia^23^. Haga et al.^22^ studied the similar negative effect of lactate on levels of total bilirubin, and hypothesized that bilirubin is a free radical scavenger consumed in neonatal HIE, and that the degree of consumption correlated with the severity of the hypoxia. Despite the combination of other biomarkers, such as lactate and LDH, UB level is useful in predicting the severity of HIE, bilirubin level measurement is also a rapid and convenient method.

This study had some limitations. It was retrospective and included a limited number of neonates with HIE to analyze the initial UB levels after admission and during the first week after birth. Therefore, further studies with larger sample sizes are required. However, because our cases were from a specific population without differences in gestational age, body weight, sex, or medical conditions other than HIE, after excluding cases with congenital anomalies, preterm birth, or genetic disorders, the bias should be reduced. Furthermore, aggressive imaging studies were not available in the HIE stage I group with favorable outcomes. However, a series of HUS could support the imaging findings, and clinical follow-up 1 year later could prove that those patients did not have significant brain parenchymal lesions.

## Conclusion

The bilirubin level in the first week is an important biomarker for clinical staging, MRI findings, seizures after discharge, and neurodevelopmental outcomes at least 1 year secondary to HIE. The peak UB level in the first week was correlated with brain MRI findings and neurodevelopmental outcomes at 1 year of age. The potential neuroprotective role for patients with HIE should be further evaluated.

### Supplementary Information

Supplementary Table 1.

## Data Availability

The datasets used and/or analysed during the current study available from the corresponding author on reasonable request.
