# Supplementary material for: Unconjugated bilirubin is correlated with the severeness and neurodevelopmental outcomes in neonatal hypoxic-ischemic encephalopathy
Source: Sci Rep. 2023 Dec 27;13:23075. doi: 10.1038/s41598-023-50399-4 (PMC10754932; doi:10.1038/s41598-023-50399-4)
Supplement: Supplementary file 1 — Supplementary Table 1. [file 41598_2023_50399_MOESM1_ESM.docx]

| Supplementary Table 1. Literature review for bilirubin correlated with neonatal hypoxic-ischemic encephalopathy. | | | | |
| --- | --- | --- | --- | --- |
| Authors | **Methods and patients** | **Results** | **Significances** | **Reference** |
| Elsadek et al., 2021 | 100 full-term with perinatal asphyxia and 50 healthy  neonates as controls. | 1. SGOT and SGPT had a positive correlation with the severity of HIE. On the 3th day, LDH rises as the stage of HIE progressed from stage 0 to stage 3. The difference in LDH among most stages of HIE was statistically significant. | Liver enzymes can be an easy early diagnostic marker to HIE. | **^25^** |
| Zou et al., 2019 | 45 infants with HIE + TH group, 44 infants with HIE + NT group and 49 infants without HIE | 1. Lower TSB in the HIE + NT group than in the controls (*P* < 0.05). 2. Not different between HIE + TH group and HIE + NT group at any time point (P>0.05). 3. Peak TSB at 96 hours: a good correlation to the results of the BSID-III (*P* = 0.02). | Bilirubin exerts a neuroprotection during the first week; low temperature not affect the antioxidant function. | **^21^** |
| Dani et al., 2018 | 40 infants with HIE and 40 infants without HIE | 1. Peak TSB and mean TSB were lower in HIE without TH and with hypothermia groups compared with control. 2. HIE + TH significantly reduced the risk of developing serum bilirubin values higher than median value (> 8.4 mg/dL). | HIE and hypothermia independently decreased TSB; low TSB in HIE could be due to hypoxic repression of HO expression and represent a defensive strategy for limiting brain injuries. | **^26^** |
| Dani et al., 2021 | Organotypic hippocampal slice cultures obtained from the brains of male and female Wistar rat pups. | 1. UB can induce neurotoxicity and oxidative stress in injured brain of term and preterm with HIE 2. Allopurinol could deserve attention as a novel pharmacological intervention for HIE. | UB abolished the neuroprotection induced by preconditioning and increased oxidative stress. These effects were restored by allopurinol. | **^23^** |

HIE indicates hypoxic-ischemic encephalopathy; TSB, total serum bilirubin; TH, therapeutic hypothermia; NT , normothermia.

TSB, total serum bilirubin; UB, unconjugated bilirubin; SGOT, serum glutamic oxaloacetic transaminase; SGPT, serum glutamic-pyruvic transaminase; HO, heme-oxygenase; BSID-III, Bayley Scales of Infant and Toddler Development, Third Edition.
